# Analysis of the benefit of anti-PD-1 monotherapy according to NGS-diagnosed genetic alterations in patients with non-small cell lung cancer

**DOI:** 10.37349/etat.2024.00283

**Published:** 2024-12-18

**Authors:** Hortense De Saint Basile, Reza Elaidi, Zineb Maaradji, Hélène Blons, Rym BenDhiab, Laure Gibault, Elizabeth Fabre

**Affiliations:** University of Salford, UK; ^1^Department of Thoracic Oncology, Georges Pompidou European Hospital, Paris Cité University, AP-HP, CARPEM, 75015 Paris, France; ^2^Department of Thoracic Oncology, Georges Pompidou European Hospital, 75015 Paris, France; ^3^Department of Molecular Biology, Georges Pompidou European Hospital, Paris Cité University, AP-HP, CARPEM, 75015 Paris, France; ^4^Department of Pathology, Georges Pompidou European Hospital, Paris Cité University, AP-HP, CARPEM, 75015 Paris, France; ^5^INSERM U970, Université Paris-Cité, 75015 Paris, France

**Keywords:** Non-small cell lung cancer, checkpoint inhibitors, NGS, mutations, biomarker, KRAS, TP53, co-mutations

## Abstract

**Aim:**

Immune checkpoint inhibitors improved the survival of advanced non-small cell lung cancer. However, only 20% of patients respond to these treatments and the search for predictive biomarkers of response is still topical. The objective of this work is to analyze the anti-PD-1 monotherapy benefit based on genetic alterations diagnosed by next generation sequencing (NGS), in advanced non-small cell lung cancer.

**Methods:**

Patients with advanced non-small cell lung cancer treated with immunotherapy were retrospectively included in this monocentric study. Clinical data, immunohistochemical expression of PD-L1 and molecular data, with a 22-genes NGS panel, were collected.

**Results:**

107 patients were included. The median age was 65 years [59; 73], 70 were men (65%), 96 had adenocarcinoma (90%), 33 were receiving a first line (31%). 54 patients had KRAS mutation (50%) and 56 had TP53 mutation (52%). The remaining mutations were present in fewer than 10 patients. There was no statistically significant differences in median of progression-free or overall survival based on KRAS-only, TP53-only or KRAS-TP53 mutations co-mutated compared to double wild-type patients (*P* = 0.46 and *P* = 0.72 respectively).

**Conclusions:**

The search for a predictive composite biomarker remains a major issue in the coming years.

## Introduction

Non-small cell lung cancer (NSCLC) is one of the most common cancers in the world and has the highest mortality rate. The last decade has seen the advent of checkpoint inhibitors and the development of molecular techniques enabling to complete and clarify histopathological classifications. The vast majority of patients can therefore benefit from extensive molecular testing at diagnosis [1], providing prognostic and predictive criteria for treatment. Immune checkpoint inhibitors (ICI) improved the survival of advanced NSCLC [2, 3]. However, only 20% of patients respond to these treatments and the search for predictive biomarkers of response is still topical. The mechanisms of response and resistance to anti-programmed death 1 (PD-1)/programmed death ligand 1 (PD-L1) drugs, and the biomarkers derived from them, can be grouped into two categories: host-centric (the study of intestinal microbiota, for example), or tumor-centric [4], which is currently the most advanced. Within the tumor itself, different types of biomarkers can be distinguish belonging to the tumor cell itself or belonging to the microenvironment, even if there is a close relationship between the two.

Alterations in intracellular signaling pathways have a direct impact on the tumor microenvironment, making the efficacy of immunotherapy uncertain. For example, Kirsten rat sarcoma viral oncogene homolog (KRAS) activation leads to the production of pro-inflammatory cytokines recruiting neutrophils and tumor-associated macrophages, which have pro-tumor properties [5]. KRAS mutations have also been reported to induce increased PD-L1 expression in NSCLC [6–8].

The objective of this work was to estimate the effect of genetic alterations on progression free survival (PFS) and overall survival (OS) in advanced NSCLC treated by checkpoint inhibitors. Analyses were restricted to monotherapy regimens considering that immunotherapy combined to chemotherapy can preclude robust conclusions on anti-PD-1/PD-L1 escape.

## Materials and methods

This monocentric, retrospective study included advanced NSCLC patients from European Georges Pompidou Hospital Paris, France, who started anti-PD-1 monotherapy between January 2015 and June 2020.

### Cohort selection, exclusion criteria, radiological definitions

Inclusion criteria were: age > 18 years, histologically confirmed diagnosis of NSCLC, locally advanced stage not accessible to local treatment or metastatic stage, treatment by anti-PD-1 monotherapy, at least one tumor evaluation, available results of tissue next generation sequencing (NGS) as described below.

Patients were excluded in case of other active neoplasm in the last 5 years, prior anti-PD-1/PD-L1 treatment; concomitant immunosuppressive therapy, treatment discontinuation prior first evaluation.

Follow-up included contrast-enhanced chest-abdominal-pelvic computer tomography (CT-scans), and brain MRI when warranted. Response was evaluated according to response evaluation criteria in solid tumors (RECIST) Version 1.1. for all patients and collected from imaging reports.

### Clinical, pathological, and radiological data

All patients treated with nivolumab or pembrolizumab for metastatic lung cancer from January 2015 to June 2020 in our center were extracted from CHIMIOPROD, a treatment prescription software. All clinical, pathological, molecular, and radiological data were extracted from electronic patient files.

### Next generation sequencing

NGS was performed routinely on tissue biopsies or surgical specimens, after preparation of a multiplex polymerase chain reaction (PCR) library including selected regions of interest from the AmpliSeq™ Colon and Lung Cancer Panel.

This selection covers over 500 hotspot mutations [KRAS, epidermal growth factor receptor (EGFR), B-Raf kinase family (BRAF), PIK3CA, AKT1, erythroblastic leukemia viral oncogene 2 (ERBB2), phosphatase and tensin homolog (PTEN), neuroblastoma RAS viral oncogene homolog (NRAS), serine-threonine kinase 11 (STK11), MAP2K1, anaplastic lymphoma kinase (ALK), discoidin domain receptor tyrosine kinase 2 (DDR2), catenin (cadherin associated protéine) beta 1 (CTNNB1), tyrosine-protein kinase Met (MET), tumor protein p53 (TP53), SMAD family member 4 (SMAD4), F-box/WD repeat-containing protein 7 (FBXW7), fibroblast growth factor receptor 3 (FGFR3), Notch homolog translocation associated 1 (NOTCH1), ERBB4, FGFR1, and FGFR2] using Ampliseq technology (Ion ampliseq library kit V2, Ion library equalizer kit, Life Technologies). Clonal amplification was performed on the Ion Chef™ System (ION P1 HI-Q CHEF, ION PI CHIP KITV3), sequencing on Ion Proton system, data analysis by Torrent Suite 4.4.3, annotation-Safir2 report tool. Variant detection threshold is 2%, minimum depth of negative results is 300X.

Patients were categorized as “mutated for TP53” in the presence of a non-synonymous mutation resulting in a non-functional protein as referenced in the IARC (International Agency for Research on Cancer) database.

PD-L1 expression was found in pathological reports. The PD-L1 score was assessed immunohistochemically, prior anti-PD-1/PD-L1 treatment, using the tumor proportion score (TPS) with Dako clone 22C3.

### Statistical analyses

The primary endpoint was OS, defined as time from ICI initiation to death whatever the cause. Alive patients were censored at the date of last follow-up. Secondary endpoints included PFS, defined by the time from ICI initiation to disease progression or death whatever the cause, objective response rate (ORR), and disease control rate (DCR). Patients who exhibited no progression were censored at the date of last follow-up.

Objective response rates were compared using Chi^2^ Log-rank test was used to compare PFS curves. The relationship between PFS/OS survival and co-mutations was investigated using a Cox regression model stratified on line of treatment and adjusted for anticipated confounders (age, smoking status, categorized PD-L1 expression). Standard diagnostics (risk proportionality, etc.) were performed to assess Cox regression applicability. Hazard ratios (HRs) were estimated with their 95% confidence intervals (95% CIs). When the relationship between the variable of interest (co-mutations or number of mutations) and the co-factors of interest (age and smoking status) was significant with at least one test, an interaction term was introduced into the model to judge its statistical significance. Covariables effect sizes in multivariable model were statistically significant if their *P*-values were < 0.05. Statistical analyses were performed using R.4.1.3.

### Ethics

The present study has been accepted and registered to the relevant institutional research (OncoHEGP: PRB-HEGP-URC NIF) and ethical committee (European Georges Pompidou Hospital) and has been conducted in accordance with the Declaration of Helsinki. All patients provided informed consent under the European Georges Pompidou Hospital approved protocol allowing collection and analysis of data.

## Results

### Patients and tumors characteristics

The charts of 204 advanced NSCLC patients were reviewed; all patients had received a first course of PD-1/PD-L monotherapy starting between January 2015 and June 2020.

**Figure 1:**
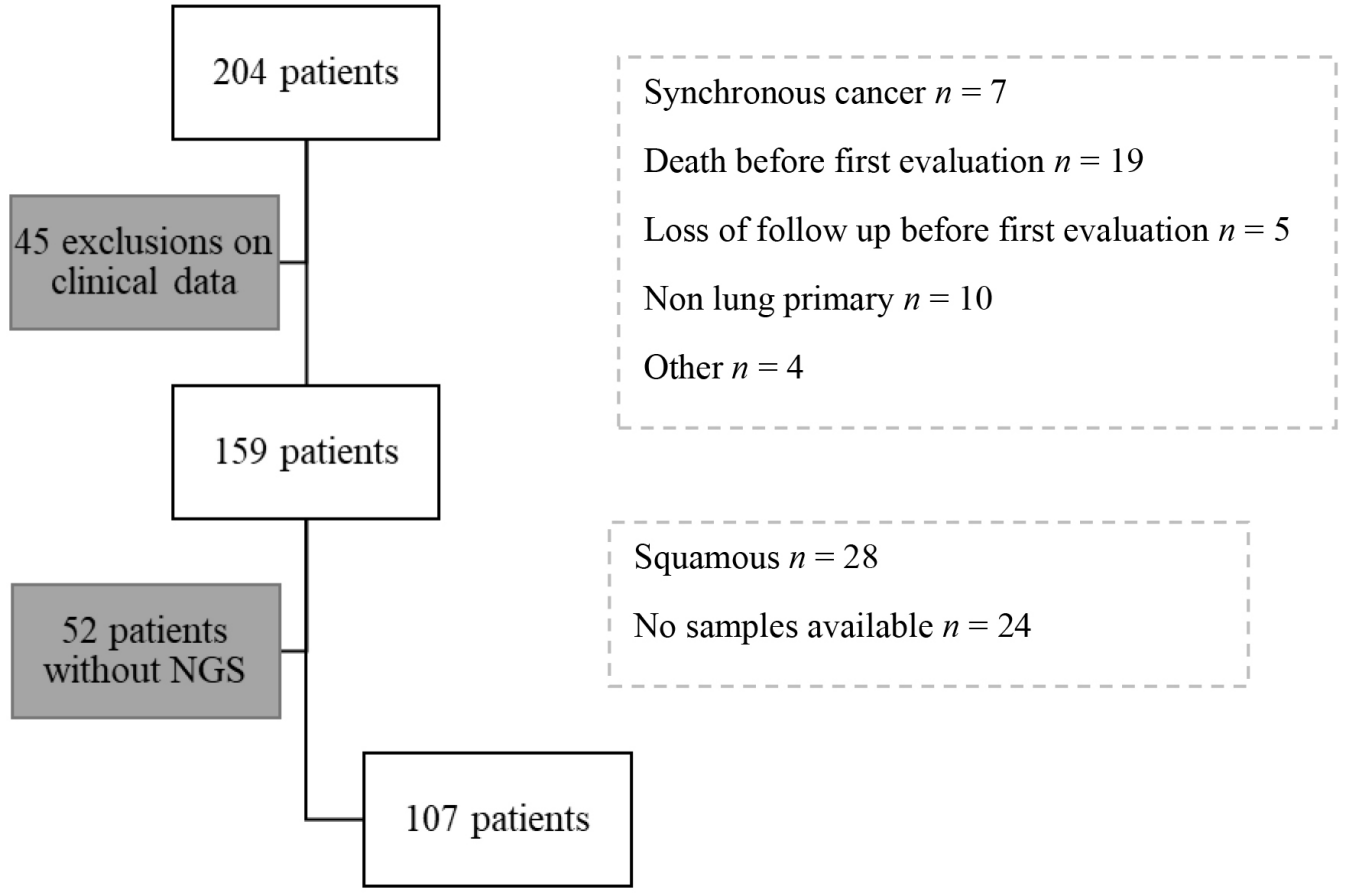
Study flowchart. NGS: next generation sequencing

Of the 204 patients identified, 45 were excluded on clinical grounds: synchronous cancer (*n* = 7), death before re-evaluation (*n* = 19), lost to follow-up before re-evaluation (*n* = 5), non-lung primary (*n* = 10), other (*n* = 4). 52 of the remaining 159 patients did not have NGS [squamous cell carcinoma *n* = 28, tumor material not available *n* = 19, sampling outside hôpital européen Georges Pompidou (HEGP) *n* = 5]. Exclusions reasons are detailed in study diagram (Figure 1).

A total of 107 patients could be analyzed (Table 1): 65% were men (*n* = 70), 91% of patients were smokers (*n* = 97). The mean smoking rate among smokers was estimated at 37 pack-years (+/–21.3). The median age at the start of immunotherapy treatment was 65 years [59; 73]. These characteristics are similar to those usually observed. 90% of patients had bronchial adenocarcinoma (*n* = 96), 7% had squamous cell carcinoma (*n* = 8), 3% had poorly differentiated or large cell cancer (*n* = 3).

**Table 1:** Baseline characteristics of patients with non-small-cell lung cancer treated with immune checkpoint inhibitors

**Variable**	**Whole cohort (*n* = 107)**
Sex	
Male	70 (65%)
Female	37 (35%)
Age	
Median	65
IQR	[59; 73]
Smoking status	
Non-smoker	9 (8%)
Smoker	97 (91%)
Unknown	1 (1%)
Histology	
Adenocarcinoma	96 (90%)
Squamous	8 (7%)
Undifferentiated	2 (1.9%)
Large cell carcinoma	1 (0.9%)
Line of treatment	
1	33 (31%)
2	57 (53%)
≥ 3	17 (16%)
PD-L1 TPS	
0%	14 (13%)
1–49%	23 (21%)
> 50%	44 (41%)
Missing	26 (24%)

PD-L1: programmed death ligand 1; ICR: confidence interval range; TPS: tumor proportion score

With regard to molecular data (Table 2), the estimated number of tumor cells per sample was acceptable, with 79% of patients having more than 20% tumor cells. This data was missing for 14% of patients. 11% of patients had no mutation found by NGS (*n* = 12), 37% of patients had one mutation, 40% two mutations, and 12% three or more mutations.

**Table 2:** Molecular data on whole cohort

**Variable**	**Whole cohort (*n* = 107)**
Tumor cells	
< 20%	7 (7%)
20–50%	45 (42%)
> 50%	40 (37%)
Missing	15 (14%)
Number of mutations	
0	12 (11%)
1	39 (37%)
2	43 (40%)
≥ 3	13 (12%)

Patients received anti-PD-1/PD-L1 in the first (31% of patients, *n* = 33), second (52%, *n* = 59), or ≥ third line (18%, *n* = 20), 83 patients had PD-L1 immunohistochemical evaluation. The median expression was 60% [2; 80]. 13% of patients were PD-L1 negative, 21% of patients had expression between 0 and 50% and 41% of patients had expression > 50%. Excluding patients treated in the first-line setting, median PD-L1 expression was 15% [2; 70].

**Figure 2:**
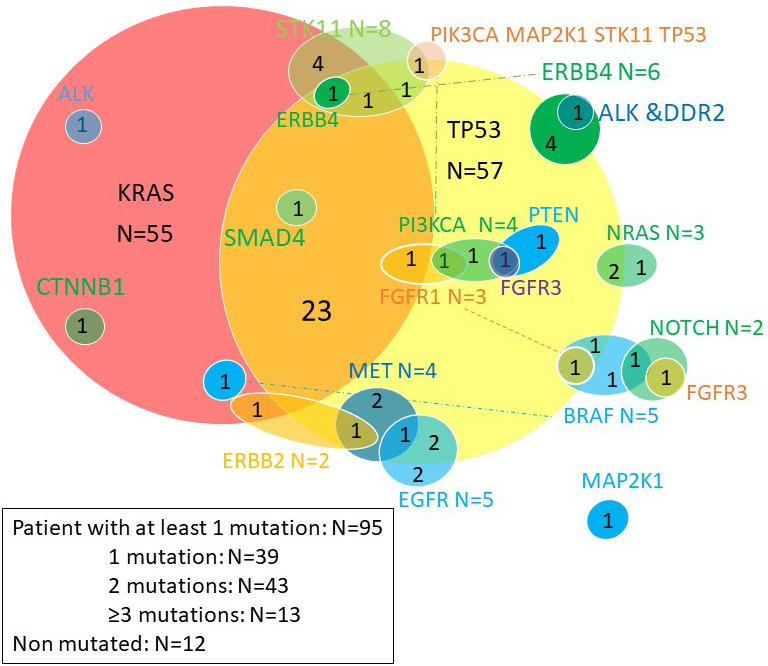
Venn diagram of somatic mutations. ALK: anaplastic lymphoma kinase; CTNNB1: catenin (cadherin associated protéine) beta 1; STK11: serine-threonine kinase 11; ERBB: erythroblastic leukemia viral oncogene; MET: tyrosine-protein kinase Met; PI3KCA: phosphatidylinositol-4,5-bisphosphate 3-kinase catalytic subunit alpha; EGFR: epidermal growth factor receptor; BRAF: B-Raf kinase family; NOTCH: Notch homolog translocation associated; NRAS: neuroblastoma RAS viral oncogene homolog; PTEN: phosphatase and tensin homolog; DDR2: discoidin domain receptor tyrosine kinase 2

The Venn diagram below (Figure 2) provides a quick overview of the distribution of mutations and co-mutations in the entire cohort. KRAS and TP53 mutations are the most common, present in 50% and 52% of patients respectively, and are associated in 23 patients. The majority of other mutations are rare, found in fewer than ten patients. KRAS mutations were G12C in 30% of cases, G12V in 26%, G12D in 11%, G12A in 7% and others (G12F, G13A, G13C) in 7%.

Molecular abnormalities in EGFR included 2 targetable mutations (L858R mutation, an exon 19 deletion), two amplifications and one duplication. BRAF mutations included 1 V600E mutation, and 4 non-targetable mutations. Molecular abnormalities in ALK were non-targetable point mutations. MET abnormalities included 3 amplifications and an exon 14 splice variant.

Four patients with squamous cell carcinoma had molecular abnormalities atypical for this histological type, suggesting a secondary adenocarcinomatous contingent: a KRAS G12C mutation, a KRAS amplification, a STK11 mutation and an ALK mutation.

Table 3 details the clinical characteristics of patients according to the two most common mutation types, KRAS and TP53. Four categories of patients were defined: double wild-type (*n* = 20), KRAS mutated (m) and TP53 wild-type (wt) (*n* = 31), KRAS wild-type (wt) and TP53 mutated (m) (*n* = 33), or double mutated (*n* = 23). The double wild-type patients were older than the other categories, and included more women and non-smokers. These clinical characteristics were all expected.

**Table 3:** Clinical characteristics according to KRAS and TP53 status

**Variable**	**KRAS-wt; TP53-wt** **(*n* = 20)**	**KRAS-m; TP53-wt** **(*n* = 31)**	**KRAS-wt; TP53-m** **(*n* = 33)**	**KRAS-m; TP53-m** **(*n* = 23)**
**Age**				
Median (IQR)	71 (± 12)	68 (± 11)	63 (± 16)	61 (± 14)
**Sex**				
Female	9 (45%)	8 (26%)	11 (33%)	9 (39%)
Male	11 (55%)	23 (74%)	22 (67%)	14 (61%)
**Smoking status**				
Non-smoker	4 (20%)	2 (6%)	2 (6%)	1 (4%)
Smoker	15 (75%)	29 (94%)	31 (94%)	22 (96%)
Missing	1 (5.0%)	0 (0%)	0 (0%)	0 (0%)
**Histology**				
Squamous	2 (10%)	0 (0%)	5 (15%)	1 (4%)
Non squamous	18 (90%)	31 (100%)	28 (85%)	22 (96%)
**Line of treatment**				
1	6 (30%)	14 (45%)	7 (21%)	6 (26%)
> 1	14 (70%)	17 (55%)	26 (79%)	17 (74%)
**PD-L1 TPS %**				
0	3 (15%)	5 (16%)	4 (12%)	2 (9%)
1–49	6 (30%)	6 (19%)	6 (18%)	5 (22%)
> 50	7 (35%)	15 (48%)	12 (36%)	10 (43%)
Missing	4 (20.0%)	5 (16.1%)	11 (33.3%)	6 (26.1%)
**Number of mutation (s)**				
Median (IQR)	0 (± 1.0)	1.0 (± 0.50)	2.0 (± 1.0)	2.0 (± 0)
**KRAS mutation**				
Other than G12C		22 (71%)		14 (61%)
G12C		9 (29%)		9 (39%)

KRAS: Kirsten rat sarcoma viral oncogene homolog; KRAS-wt: KRAS wild type; KRAS-m: KRAS mutated; TP53: tumor protein p53; TP53-wt: TP53 wild type; TP53-m: TP53 mutated; PD-L1: programmed death ligand 1; TPS: tumor proportion score; ICR: confidence interval range

### Analysis of PFS

Median follow-up was 40.6 months (33.8; 56.7). Median PFS for patients treated in 1st line was 8.55 months [95% CI 2.73; 17.24] and median OS 23.9 months [95% CI 14.1; NA]. Median PFS for patients treated in 2nd line or more was 3.2 months [95% CI 2.2; 6.2].

Median PFS for KRAS-mutated patients was 3.5 months [95% CI 2.2; 12.0] vs. 3.8 months [95% CI 2.4; 8.8] for wild-type patients (Table 4). Median PFS for TP53-mutated patients was 3.8 months [95% CI 2.2; 12] vs. 3.7 months [95% CI 2.3; 8.8] for wild-type patients.

**Table 4:** Median PFS according to KRAS and TP53

**KRAS and TP53 molecular profile**	***N***	**Events**	**PFS median**	**0.95LCL**	**0.95UCL**
KRAS-wt; TP53-wt	20	20	3.3	1.8	10.9
KRAS-m; TP53-wt	31	27	3.7	2.3	14.0
KRAS-wt; TP53-m	33	25	4.1	2.2	14.1
KRAS-m; TP53-m	23	21	3.0	1.9	17.2

KRAS: Kirsten rat sarcoma viral oncogene homolog; TP53: tumor protein p53; *N*: number of patients; KRAS-wt: KRAS wild type; KRAS-m: KRAS mutated; TP53-wt: TP53 wild type; TP53-m: TP53 mutated; PFS: progression free survival; LCL: lower control limit; UCL: upper control limit

In univariate analyses, there was no significant impact of KRAS mutations on PFS (*P* = 0.67). Similarly, there was no significant effect of TP53 mutations on PFS (*P* = 0.44).

**Figure 3:**
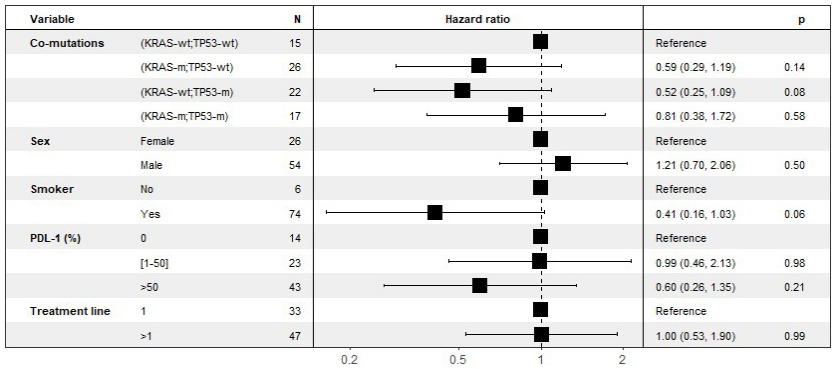
Multivariate analyses of the effect of co-mutations on PFS. Stratified by line of treatment (last column: *P*-value for statistical significance). KRAS: Kirsten rat sarcoma viral oncogene homolog; KRAS-wt: KRAS wild type; KRAS-m: KRAS mutated; TP53: tumor protein p53; TP53-wt: TP53 wild type; TP53-m: TP53 mutated; PFS: progression free survival

In order to analyze the participation of each subgroup and to eliminate the impact of confounding factors (gender, smoking status, PD-L1 expression), a multivariate analyses was then performed multivariate to analyze the effect of co-mutations, stratified by line of treatment (Figure 3).

There was no effect of gender or treatment line on PFS. In association with co-mutations, smoking had a protective effect on PFS, and PD-L1 expression had a protective effect on PFS for those over 50%.

### Analysis of OS

Median OS for patients treated in 1st line was 23.9 months [95% CI 14.1; NA], and for patients treated in 2nd line or more was 21.4 months [95% CI 11.1; 33.8].

Median OS for KRAS-mutated patients was 19.4 months [95% CI 12.6; 47.0] vs. 22.4 months [95% CI 10.8; 42.2] for wild-type patients (Table 5). In univariate analyses, there was no significant impact of KRAS mutations on OS (*P* = 0.67). Median OS for TP53-mutated patients was 21.4 months [95% CI 12; 42.5] vs. 23.9 months [95% CI 10.8; 42.2] for wild-type patients. Similarly, there was no significant effect of TP53 mutations on OS (*P* = 0.76).

**Table 5:** Median OS according to KRAS and TP53 molecular profile

**KRAS and TP53 molecular profile**	***N***	**Events**	**Median OS (95% CI)**	**0.95LCL**	**0.95UCL**
KRAS-wt; TP53-wt	20	15	16.8 (9.3–NR)	9.3	NR
KRAS-m; TP53-wt	31	18	30.4 (8.8–NR)	8.8	NR
KRAS-wt; TP53-m	33	20	23.0 (11.1–NR)	11.1	NR
KRAS-m; TP53-m	23	33	19.0 (11.1–NR)	11.1	NR

TP53: tumor protein p53; KRAS: Kirsten rat sarcoma viral oncogene homolog; *N*: number of patients; KRAS-wt: KRAS wild type; KRAS-m: KRAS mutated; TP53-wt: TP53 wild type; TP53-m: TP53 mutated; OS: overall survival; NR: not reached. LCL: lower control limit; UCL: upper control limit

**Figure 4:**
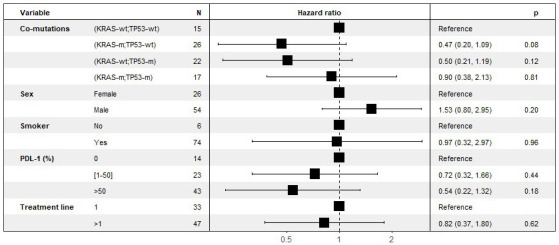
Multivariate analyses of the effect of co-mutations on OS, stratified by line of treatment. KRAS: Kirsten rat sarcoma viral oncogene homolog; KRAS-wt: KRAS wild type; KRAS-m: KRAS mutated; TP53: tumor protein p53; TP53-wt: TP53 wild type; TP53-m: TP53 mutated; OS: overall survival

PD-L1 expression had a linear effect on OS for those over 1% (Figure 4).

### Overview of the impact of co-mutations on benefit to anti-PD-1/PD-L1

**Figure 5:**
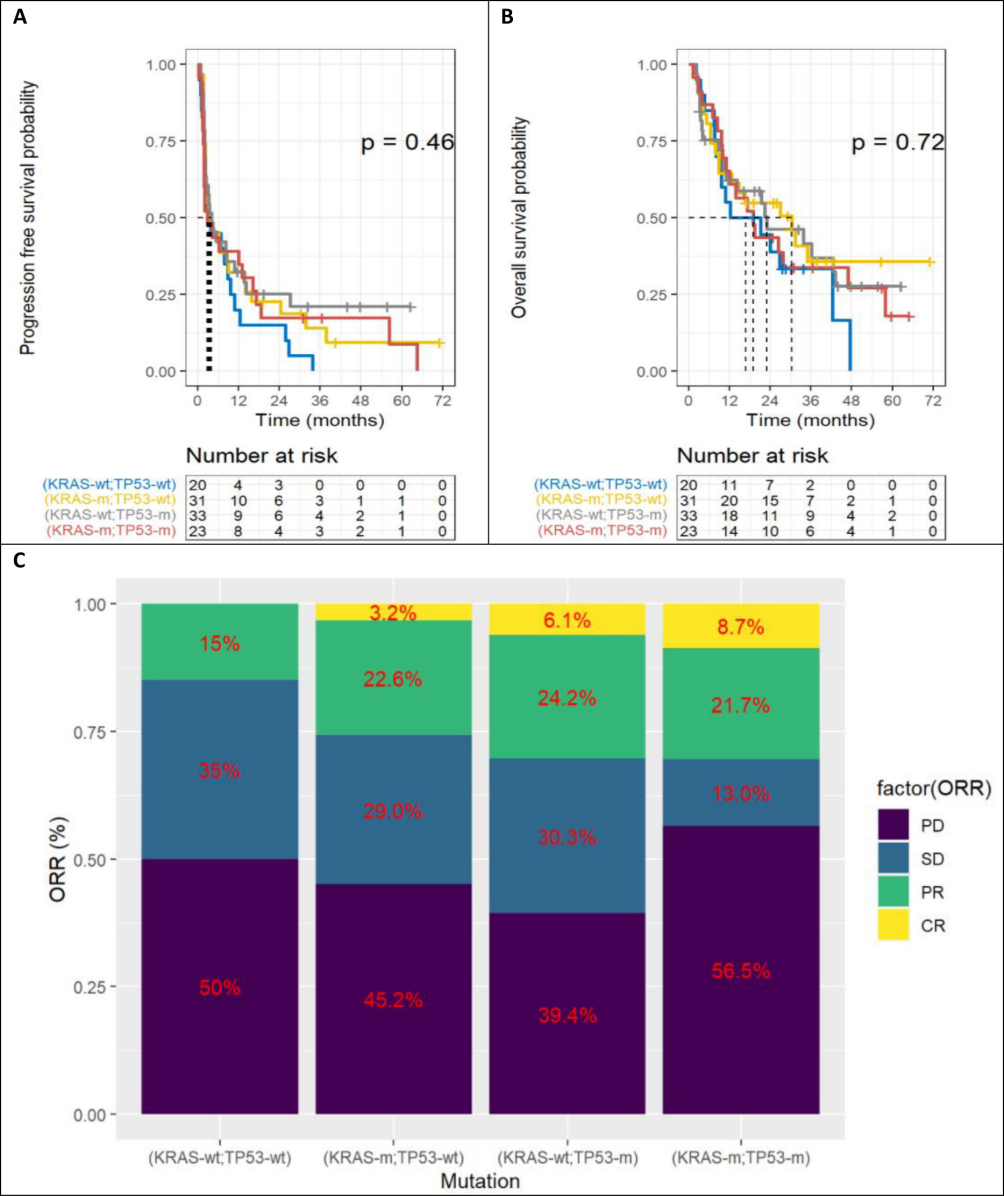
Benefit according to KRAS and TP53. A. Progression free survival; B. overall survival; C. objective response rate (ORR). TP53: tumor protein p53; KRAS: Kirsten rat sarcoma viral oncogene homolog; KRAS-wt: KRAS wild type; KRAS-m: KRAS mutated; TP53-wt: TP53 wild type; TP53-m: TP53 mutated; PD: progressive disease; SD: stable disease; PR: partial response

The Figure 5 represents Kaplan-Meier curves below represent PFS (Figure 5A), OS (Figure 5B) and ORR (Figure 5C) according to the four categories of patients, depending on KRAS and TP53 mutations. There is no statistically significant difference. However, there is a trend for double wild-type patients to have poorer OS.

No statistically significant results were found, given the small size of the cohort, but there was a non-zero effect of the isolated TP53 mutation on PFS (HR 0.52 [95% CI 0.25; 1.1] *P* = 0.082) and OS (HR 0.50 [95% CI 0.21; 1.2] *P* = 0.117). Similarly, there was a non-zero effect of isolated KRAS mutation, especially on OS (HR 0.47 [95% CI 0.20; 1.1] *P* = 0.078). On the other hand, there was no benefit in the case of KRAS and TP53 double mutation.

## Discussion

This cohort had the usual clinical and molecular features of NSCLC. This is a “real life” study with no statistically significant differences in PFS or OS based on KRAS-only, TP53-only or KRAS-TP53 co-mutated compared to double wild-type patients (*P* = 0.46 and *P* = 0.72 respectively). Multivariate analyses showed trends in favor of isolated KRAS and TP53 mutations, with benefit magnitudes in line with the literature.

Two meta-analyses of over 500 patients, incorporating data from three registration trials (Checkmate 057, POPLAR and OAK), found an OS benefit to the use of ICI in KRAS mutated patients vs. chemotherapy in non-mutated patients, with a HR of 0.64 (95% CI: 0.43–0.96), *P* = 0.03 [9, 10]. PFS was not analyzed. A meta-analysis of 6 registration trials of immunotherapies in first-line (as monotherapy or in combination with chemotherapy) and second-line monotherapy, found significantly longer OS in KRAS-mutated patients compared with wild-type (*P* = 0.001) [11]. In a retrospective cohort of 1,127 first-line patients with PD-L1 expression above 50%, KRAS mutations were significantly associated with better OS on pembrolizumab (21.1 vs. 13.6 months; *P* = 0.03) [12]. Finally, a meta-analysis of prospective single-agent immunotherapy trials found an ORR of 21.9% (95% CI: 14.0–30.9) in KRAS mutated patients (*n* = 198) vs. 17.4% (95% CI: 11.3–24.5) in wild-type patients (*n* = 452) [13].

In the IMMUNOTARGET study, progression-free survival was unaffected by subtype of alteration (G12C vs. others), even though some are known to be more related to smoking [14]. Data on the response rates by mutation type weren’t available. However, in first-line treatment, patients with a KRAS G12C mutation benefit, compared with other KRAS mutations or wild-type status, in terms of objective response rate (63.3% vs. 42.7% *P* = 0.06), PFS (19.8 months vs. 6.2 months, *P* < 0.001) and OS (NA vs. 23.4 months *P* = 0.08) [15].

In a French cohort of 72 patients, median OS in mutated TP53 patients was 18.1 months (95% CI: 6.6–not reached) vs. 8.1 months (95% CI 2.2–14.5, HR = 0.48; 95% CI 0.25–0.95, *P* = 0.04) in the wild-type TP53 group [16]. There was no metanalyse evaluating the benefit of checkpoint inhibitors according to TP53 mutation status. However, mutations in this gene are usually associated with resistance to conventional anti-cancer therapies [17]. Analysis of the impact of TP53 mutations on the tumor microenvironment reveals that isolated TP53 mutations identify a tumor subgroup with the highest CD8 lymphocyte infiltration and PD-L1 expression [18]. TP53 mutations significantly increase activated effector T cell infiltration and interferon γ signature [8, 19].

TP53 effect on treatment response may be mediated by its role as a “genome gatekeeper”, with TP53 mutations having been associated with a higher mutational load in NSCLC [20]. NGS analysis is not binary: each type of molecular variant must also be integrated [21]. With regard to TP53, not all mutations are equal in predicting efficacy in patients treated with ICI. First of all, R175H, R248W, R248Q, R249S, R273H, R273L, and R282W can be considered as gain of function (GOF) and all other TP53-m as non-GOF [22]. Moreover, in a multicenter data analysis, missense and nonsense mutations were associated with higher mutational loads and neoantigen levels, and also contributed to a deficit in DNA damage repair. However, only TP53 missense mutations showed increased PD-L1 expression. This unique subgroup is associated with clinical benefit from ICI [23]. This heterogeneity of TP53 mutations calls for special attention when assessing TP53 status as a biomarker, which is found in several tumor types [24].

Co-mutation analysis also has an impact. Several studies found that patients with a KRAS and TP53 co-mutation had a greater clinical benefit on immunotherapy [19, 25, 26]. In patients treated in the first line with pembrolizumab monotherapy, the presence of a KRAS G12C mutation associated with a TP53 co-mutation is associated with a major clinical benefit (ORR 100.0%, PFS 33.3 months, OS NA) [15], mediated by highly active interferon gamma signaling in a proinflammatory tumor microenvironment [26]. TP53/KRAS-mutated tumors show increased PD-L1 expression, as well as increased mutational load. In another publication, mutations in TP53, STK11 and EGFR are major determinants of the intra-tumoral immune profile and PD-L1 expression by tumor cells [18].

Longer survival is observed in patients with TP53 mutation without STK11 or EGFR mutation (HR = 0.32; 95% CI, 0.16–0.63, *P* = 0.001) compared to patients with STK11 or EGFR mutation [18, 27]. Same for STK11 mutations associated with KRAS are associated with very poor prognosis [28]. In our cohort, the proportion of patients with this type of mutations is too low to be analyzed.

The low incidence of some genetic alterations precludes robust data on their predictive impact. Some studies have thus pooled rare mutations to study their prognostic value [29] but there are few robust studies concerning their predictive value.

Moreover, the molecular presentation of a cancer is heterogeneous and dynamic, as is its tumor microenvironment [30]. Somatic molecular alterations, as well as the tumor environment, are not a static criterion, but can evolve over time, spontaneously, or under therapeutic pressure with cytotoxic chemotherapies for example, and notably alkylating agents such as platinum salts widely used in NSCLC, or targeted therapeutics. Various mechanisms are involved, such as induction of class I and II molecules, reduction of the regulatory T-cell infiltrate, enhancement of CD8 LT cytotoxicity and increased secretion of IFN γ [31]. After anti EGFR treatment, one study found that the proportion of patients with PD-L1 expression level > 50% increased from 14% to 28% after EGFR-TKI (*P* = 0.001) [32]. These results had an impact on the PFS under subsequent immunotherapy (7.1 months vs. 1.7 months, *P* = 0.0033), and two of the five patients with increased PD-L1 expression had a PFS > 6 months. Similarly, mutational load tended to be higher after treatment [33], so studies combining immunotherapy with anti-EGFR therapies look promising [28, 34].

Finally, repeated tissue sampling is invasive, costly and sometimes non-contributory. For this reason, NGS analysis of circulating tumor DNA is increasingly being developed. The value of repeat NGS at different times in the course of tumor disease can thus be discussed.

This work has some limitations. Firstly, this is a retrospective study, with the selection bias inherent in this type of study, and the results must therefore be interpreted with caution. The limited size of the sample is partly due to the retrospective nature of the study with missing data and lost of follow up, and also due to the lack of biological samples available for NGS. The age of certain samples and the lack of available equipment have limited this work. As most of the mutations were rare, analyses were made on a small number of patients. This results in a lack of power, especially in subgroup analysis. The time between the sample on which PD-L1 and NGS were performed and the start date of immunotherapy is heterogeneous, some having had it just before the start of treatment, others having much older samples with intercurrent treatments, which may result in genomic and environmental tumor changes (> 6 months: 46 patients; > 12 months: 27 patients). However, this is a real-life study, and the multiplication of samples and techniques is not systematically feasible. In addition, the distribution of patients was heterogeneous, with some patients treated in the first line and therefore all having PD-L1 > 50%, and others in the second line and beyond. Adjustment for PD-L1 status eliminates this confounding bias.

This study founds PFS data similar to those in the literature, with a median PFS of 3.5 months, but OS was much greater than in the literature. Exploring data, this atypical survival mainly concerns patients treated in the third line and beyond, with a median OS of 26 months. The number of patients in this subgroup is small, and includes a few with particularly prolonged survival. Indeed, the limitations of this study design and sample size necessitate cautious interpretation and these results must be validated in larger cohorts.

This study only included patients treated with immunotherapy in monotherapy, and conclusions cannot be extended to patients treated with combination with chemotherapy [35].

In conclusion, this study contributes to the evolving landscape of biomarker research in NSCLC immunotherapy. While the findings regarding KRAS and TP53 mutations are intriguing, the limitations of the study design and sample size necessitate cautious interpretation. Future prospective studies with larger cohorts and more comprehensive molecular profiling are warranted to validate these results and potentially uncover more robust predictive biomarkers for anti-PD-1 therapy response in NSCLC patients.

## Abbreviations

95% CIs: 95% confidence intervals
ALK: anaplastic lymphoma kinase
BRAF: B-Raf kinase family
CTNNB1: catenin (cadherin associated protéine) beta 1
DDR2: discoidin domain receptor tyrosine kinase 2
EGFR: epidermal growth factor receptor
ERBB: erythroblastic leukemia viral oncogene
FBXW7: F-box/WD repeat-containing protein 7
FGFR3: fibroblast growth factor receptor 3
GOF: gain of function
HEGP: hôpital européen Georges Pompidou
HRs: hazard ratios
ICI: immune checkpoint inhibitors
KRAS: Kirsten rat sarcoma viral oncogene homolog
m: mutated
MET: tyrosine-protein kinase Met
NGS: next generation sequencing
NOTCH1: Notch homolog translocation associated 1
NRAS: neuroblastoma RAS viral oncogene homolog
NSCLC: non-small cell lung cancer
ORR: objective response rate
OS: overall survival
PCR: polymerase chain reaction
PD-(L)1: programmed death (ligand) 1
PD: progressive disease
PFS: progression free survival
PIK3CA: phosphatidylinositol-4,5-bisphosphate 3-kinase catalytic subunit alpha
PR: partial response
PTEN: phosphatase and tensin homolog
RECIST: response evaluation criteria in solid tumors
SD: stable disease
SMAD4: SMAD family member 4
STK11: serine-threonine kinase 11
TDM: tomodensitometrie
TP53: tumor protein p53
TPS: tumor proportion score
wt: wild-type

## Declarations

### Availability of data and materials

The raw data supporting the conclusions of this manuscript will be made available by the authors, without undue reservation, to any qualified researcher.

### Copyright

© The Author(s) 2024.
